# Deciphering the interplay between serum urate and bone mineral density: insights from observational and genetic perspectives

**DOI:** 10.1080/15502783.2026.2662625

**Published:** 2026-04-22

**Authors:** Rong Xiang, Yangdan Zhong, Xunying Zhao, Qin Deng, Jiaojiao Hou, Linna Sha, Yang Qu, Mingshuang Tang, Jiangbo Zhu, Sirui Zheng, Tao Han, Jinyu Zhou, Ting Yu, Bin Yang, Xin Song, Maoyao Xia, Mengyu Fan, Chenglin Tao, Xia Jiang

**Affiliations:** a Department of Nutrition and Food Hygiene, West China School of Public Health and West China Fourth Hospital, Sichuan University, Chengdu, Sichuan, People's Republic of China; b Department of Epidemiology and Biostatistics and West China-PUMC C. C. Chen Institute of Health, West China School of Public Health and West China Fourth Hospital, Sichuan University, Chengdu, Sichuan, People's Republic of China; c West China School of Public Health and West China Fourth Hospital, Sichuan University, Chengdu, Sichuan, People's Republic of China; d Department of Clinical Neuroscience, Center for Molecular Medicine, Karolinska Institutet, Solna, Stockholm, Sweden

**Keywords:** Urate, bone mineral density, phenotypic association, genetic correlation, genome-wide cross-trait analysis, Mendelian randomization

## Abstract

**Background:**

Urate commonly influences bone metabolism due to its antioxidant properties, yet this relationship remains inadequately understood. Our study aims to investigate both the phenotypic and genetic relationships between urate and heel estimated bone mineral density (eBMD), a proxy marker for osteoporosis.

**Methods:**

Leveraging UK Biobank individual-level data and summary statistics from the hitherto largest genome-wide association studies, we evaluated phenotypic associations using linear regression and restricted cubic splines (*N* = 424,105), then explored genetic correlations, causality, and pleiotropy through genome-wide cross-trait analyses (*N*_urate_ = 355,426; *N*_eBMD_ = 426,824).

**Results:**

Observational analysis suggested a significant linear association (*β* = 0.008, *P* = 3.35×10^−10^) and an inverted U-shaped relationship (*P*_non-linear_ < 0.001) between urate and eBMD. Genome-wide genetic correlation was significant (*r*_g_ = 0.09, *P* = 2.01×10^−7^), corroborated by six local signals at chromosomes 7, 9, 10, 14, 17, and 20. Cross-trait meta-analysis identified 237 pleiotropic loci, including 28 novel loci with five showing colocalization (*PPH4* > 0.90). Gene-based analysis identified 278 shared genes. Transcriptome-wide association study revealed 150 shared genes, primarily enriched in tissues such as the tibial artery, fibroblasts, left ventricle, tibial nerve, and thyroid. One-sample Mendelian randomization demonstrated a causal effect of urate on eBMD (*β* = 0.038, *P* = 2.26×10^−21^), as well as a non-linear causal relationship (*P*_non-linearity_ = 3.89×10^−3^; *P*_heterogeneity_ = 0.02).

**Conclusions:**

Our findings support phenotypic and genetic relationships between urate and eBMD, highlighting the etiological role of urate in osteoporosis and offering potential strategies for reducing osteoporosis risk.

## 1. Introduction

Bone remodelling is a dynamic process essential for maintaining bone integrity and mineral homeostasis, regulated by factors like oxidative stress [1,2]. Disruptions in this process lead to reduced bone mineral density (BMD) and impaired microarchitecture—key indicators of osteoporosis—increasing the risk of fragility fractures and imposing substantial health burdens [3].

Urate (uric acid), the end product of purine metabolism, contributes up to 55% of extracellular antioxidant capacity [4] and is believed to protect bone metabolism under physiological conditions [5]. A meta-analysis of 19 studies involving 55,859 participants found a positive association between urate levels and BMD (standardised mean difference = 0.25–0.29; *I*^*2*^ = 33%–71%) [6]. However, other studies reported inconsistent findings, including no association [7,8] or even a negative correlation [9]. Most evidence is limited by small sample sizes, cross-sectional designs, a focus on Asian populations (particularly China), and a lack of non-linear associations, emphasising the need for large-scale, diverse data to investigate both linear and non-linear relationships between urate and BMD.

Inconsistencies in observational studies may also arise from reverse causation and confounding factors. To mitigate these, Mendelian randomisation (MR) is often employed to infer causal relationships, using single-nucleotide polymorphisms (SNPs) as instrumental variables (IVs). Both urate and BMD have substantial genetic heritability—urate around 16% with 363 SNPs [10], and heel estimated BMD (eBMD) about 38% with 1,103 SNPs [11]. Although dual-energy X-ray absorptiometry (DXA)-derived BMD at the lumbar spine or total hip remains the clinical standard for osteoporosis diagnosis, eBMD is widely used in large-scale genetic studies because of its larger available genome-wide association study (GWAS) sample size and its established relevance to osteoporosis and fracture risk [11,12]. However, no MR has explored the urate–eBMD causal relationship. Four existing MRs failed to demonstrate a causal role of urate in osteoporosis or BMD at the lumbar spine (L1–L4), femoral neck, or total hip [13–16], limited by a small number of IVs (ranging from 5 to 26) and restricted sample sizes (1,322 Chinese Han [13] and 2,501 Americans [16]).

Pleiotropy (i.e., a single variant affects both traits) may also contribute to the phenotypic link between urate and eBMD. Evidence suggests that common biological mechanisms, such as oxidative stress, may underlie this relationship [17,18]. Furthermore, a genome-wide gene-environmental interaction analysis of 237,799 participants from the UK Biobank (UKB) identified 17 significant SNP-by-urate interactions for eBMD, including rs145344540 in *PDE11A* and rs78485379 in *DKK2* [19], implying biological connections. Despite advances in genetic methods and the availability of large-scale GWASs, no study has systematically explored the shared genetic basis between urate and eBMD.

**Figure 1.**
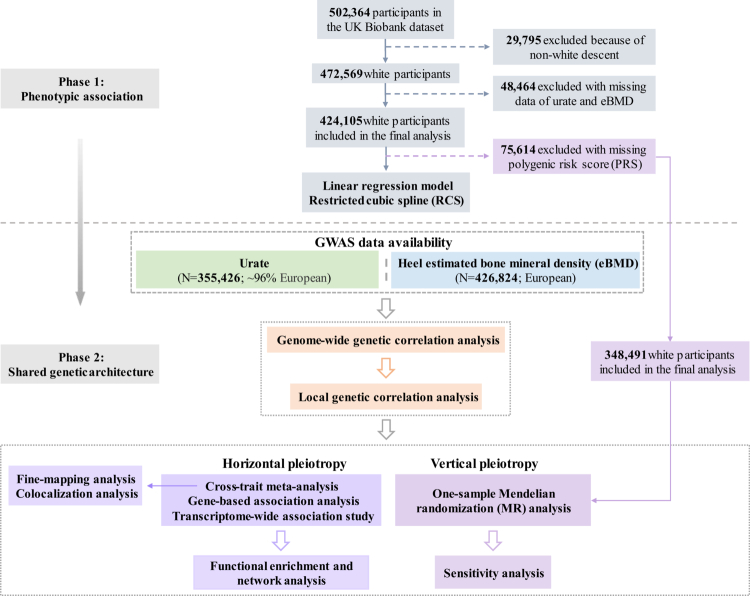
Flowchart of the overall study design. Leveraging individual-level data from the UK Biobank and summary-level statistics from the hitherto largest GWASs, both observational and genetic analyses were conducted to characterise the phenotypic associations and shared genetic architecture between urate and eBMD. eBMD: heel estimated bone mineral density; GWAS: genome-wide association study.

To comprehensively investigate the phenotypic and genetic associations between urate and eBMD, we conducted both observational and genome-wide cross-trait analyses. Phenotypic associations were assessed using linear regression and restricted cubic splines. Genetic correlation analyses were then performed to identify shared genetic bases, which were further dissected into causality and pleiotropy. Causality was explored through one-sample MR analysis to infer causal relationships, while pleiotropy was explored by identifying shared loci, genes, and gene-tissue pairs using cross-trait meta-analysis, gene-based analysis, and transcriptome-wide association study (TWAS), respectively. An overview of the study design is provided in Figure 1.

### 1.1. Data sources and methods

The Strengthening the Reporting of Observational Studies in Epidemiology (STROBE) and STROBE-MR checklists are provided in **Additional File 1**.

### 1.2. UK Biobank data

The UK Biobank is a prospective cohort study comprising 502,364 adults aged 40–69 years, with extensive phenotypic and genetic data (accessed under Application #99713). Serum urate was measured using the uricase–peroxidase aminophenazone method on a Beckman Coulter AU5800, and eBMD was estimated from the calcaneal Quantitative Ultrasound Index using a Sahara Clinical Bone Sonometer. The present study was based on baseline data. A total of 424,105 white European participants with complete baseline measurements of urate and eBMD were included.

### 1.3. GWAS summary statistics

Summary statistics were obtained from publicly available GWAS datasets. The hitherto largest GWAS for serum urate involved a meta-analysis of 355,426 participants (approximately 96% European ancestry) from the UKB 10, identifying 363 urate-associated index SNPs (*P* < 5 × 10^−9^). For eBMD, the hitherto largest GWAS analysed 426,824 European participants aged 39–74 years from the UKB [20], identifying 1,103 eBMD-associated signals (*P* < 6.6 × 10^−9^). We applied reported IVs, with *F*-statistic < 10 indicating weak instruments [21].

### 1.4. Statistical analyses

#### 1.4.1. Observational analysis

Statistical analyses were performed using R software (version 4.2.3), with *P*-values < 0.05 considered statistically significant. Descriptive statistics summarised continuous variables as means ± standard deviations or medians (interquartile range), and categorical variables as percentages. Linear regression and restricted cubic splines (RCSs) with four knots were employed to assess linear and non-linear associations, respectively. Four models were applied: (i) Model 1 adjusted for age, sex (total participants only), assessment centre, and the top 20 genetic principal components; (ii) Model 2 further adjusted for body mass index, education, Townsend deprivation index, income, smoking status, alcohol consumption, physical activity, and supplementation with calcium, vitamin D, and vitamin C; (iii) Model 3 additionally adjusted for hormone replacement therapy (female participants only), disease status (diabetes, hypertension, hyperlipidemia), and medication use (including bisphosphonates, corticosteroids, diuretics, beta-blockers, allopurinol, febuxostat, and benzbromarone); (iv) Model 4 (primary model) further included serum calcium, vitamin D, alkaline phosphatase, C-reactive protein, and estimated glomerular filtration rate (CKD-EPI).

#### 1.4.2. Genetic correlation analysis

A genome-wide genetic correlation analysis was performed using linkage disequilibrium (LD) score regression (LDSC) [22] to estimate the average genetic correlation (*r*_*g*_) shared between two traits (ranging from −1 to + 1), with *P* < 0.05 indicating statistical significance. As LDSC may fail to detect local signals, local genetic correlations were estimated in approximately 2,353 pre-defined independent LD blocks (average length: 1.6 centimorgans) using SUPERGNOVA [23], with Bonferroni correction for multiple testing (*P* < 0.05/2353). Both LDSC and SUPERGNOVA can provide relatively robust estimates of genetic correlation in the presence of sample overlap.

#### 1.4.3. Cross-trait meta-analysis

The cross-phenotype association analysis (CPASSOC) [24] was performed to identify pleiotropic loci affecting both traits, with *S*_*Het*_ used to assess heterogeneous effects. A trait correlation matrix was estimated using LD-pruned approximately null SNPs and incorporated into the CPASSOC analysis to account for correlation between the two traits, including that induced by sample overlap. Independent loci were obtained using the “clumping” function of PLINK (version 1.9) with the following parameters: *--clump-p1 5e-8 --clump-p2 1e-5 --clump-r2 0.2 --clump-kb 500* [25]. Significant pleiotropic SNPs were defined as those with *P*_single-trait_ < 1 × 10^−5^ and *P*_CPASSOC_ < 5 × 10^−8^. These SNPs were further categorised as “Known”, “Single-trait driven”, “LD-tagged”, and “novel” shared SNPs. The novel shared SNPs are neither driven by both traits (*P*_urate_ ≥ 5 × 10^−9^ and *P*_eBMD_ ≥ 6.6 × 10^−9^) nor in LD (*r*^2^ < 0.2) with index SNPs identified in the original single-trait GWASs. The Ensembl Variant Effect Predictor (VEP) was used for annotation.

#### 1.4.4. Fine-mapping and colocalization analysis

A 99% credible set of causal variants within 250 kb of each index SNP was identified using FM-summary to account for complex LD patterns [26], a Bayesian fine-mapping algorithm that maps the primary signal and generates posterior inclusion probabilities for each variant.

To determine whether the same variants drive two GWAS signals or if distinct variants are close, colocalization analysis was performed using Coloc [27]. Coloc calculates posterior probabilities for five hypotheses: H0 (no association); H1/H2 (association with one trait only); H3 (association with both traits, two distinct SNPs); H4 (association with both traits, one shared SNP). We extracted summary statistics for variants within 250 kb of the index SNP at each shared locus and calculated the posterior probability for H4 (*PPH4*). A locus was considered colocalized if *PPH4* > 0.90.

#### 1.4.5. Gene-based association analysis

To aggregate all SNPs within genes and quantify their joint association with the phenotype, a gene-level analysis was conducted using Multi-marker Analysis of GenoMic Annotation (MAGMA) [28]. The annotation was based on approximately 19,427 protein-coding genes (NCBI build 37.3), utilising the 1KGP Phase 3 panel to identify independent significant signals. A Bonferroni correction was applied for multiple testing (*P* < 0.05/19427). Significant genes from both traits were then merged to identify shared genes.

#### 1.4.6. Transcriptome-wide association study

To identify genes whose expression patterns across specific tissues suggest associations between two traits, a transcriptome-wide association study (TWAS) was performed using FUSION [29]. We integrated GWAS summary data with expression weights across 49 tissues from the Genotype-Tissue Expression (GTEx, version 8) dataset, analysing one tissue-trait pair at a time. A Bonferroni correction was applied for multiple testing within each trait, and joint/conditional tests were used to identify independent gene-tissue pairs. Shared gene-tissue pairs were defined by intersecting the results across traits.

#### 1.4.7. Functional enrichment and network analysis

To elucidate the functional roles of significant shared genes identified from CPASSOC, MAGMA, and TWAS, we used STRING (version 12.0) to construct a protein-protein interaction (PPI) network, and hub genes were identified by calculating the maximal clique centrality (MCC) using cytoHubba in Cytoscape (version 3.10.3). Kyoto Encyclopaedia of Genes and Genomes (KEGG) [30] and Gene Ontology (GO) [31] analyses were performed with the R package clusterProfiler, using a false discovery rate (FDR)-corrected *P*-value < 0.05 for statistical significance.

#### 1.4.8. One-sample Mendelian randomisation analysis

To explore the potential causal relationship between urate and eBMD, we performed a one-sample MR analysis using an unweighted polygenic risk score (PRS) derived from 363 urate-associated SNPs [10]. An unweighted PRS was adopted as a conservative instrumental variable strategy to reduce reliance on potentially inflated SNP effect estimates, given the lack of full independence between the SNP discovery source and the analytic sample [32]. Using this PRS as the instrumental variable, we applied the two-stage least squares (2SLS) method to estimate the causal effect of urate on eBMD in all participants and separately in women and men, adjusting for age, sex (total participants only), assessment centre, genotyping batch, and the top 20 genetic principal components. The plausibility of the instrumental variable assumptions was assessed using the first-stage PRS–urate association, tests of association between the PRS and measured baseline covariates, and negative control outcome analyses [33–35]. To further investigate potential non-linear causality, we applied a doubly-ranked stratification method [36], dividing the population into ten strata by instrument and urate level within each stratum. For each stratum, linear MR estimates were calculated using the ratio method to obtain localised average causal effects (LACEs), which were meta-regressed against the mean urate levels and fitted by a fractional polynomial model. Additional adjustments for age-squared, age-by-sex interaction, and age-squared-by-sex interaction were incorporated.

## 2. Results

### 2.1. Phenotypic association

**Figure 2.**
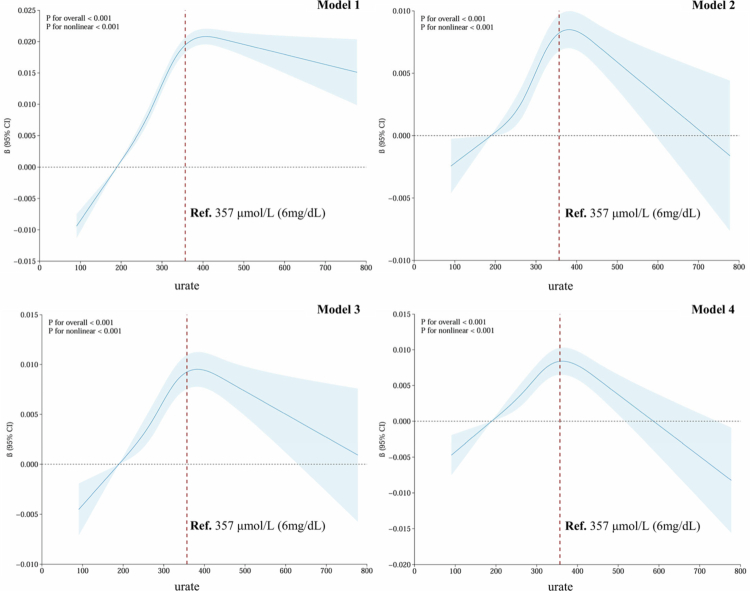
Non-linear association between urate and eBMD. Restricted cubic spline (RCS) curves were used to depict the non-linear phenotypic association between serum urate and eBMD. The solid blue lines represent adjusted *β* coefficients, and the shaded areas indicate the corresponding 95% *CIs*. The horizontal dashed line indicates *β* = 0, and the vertical red dashed line marks the reference (**Ref.**) value of 357 μmol/L (6 mg/dL), a threshold associated with potential hyperuricemia and an elevated long-term risk of gout. Model 1 adjusted for age, sex, assessment centre, and the top 20 genetic principal components; Model 2 further adjusted for body mass index, education, Townsend deprivation index, income, smoking status, alcohol consumption, physical activity, and supplementation with calcium, vitamin D, and vitamin C; Model 3 additionally adjusted for hormone replacement therapy (female participants only), disease status (diabetes, hypertension, hyperlipidemia), and medication use (including bisphosphonates, corticosteroids, diuretics, beta-blockers, allopurinol, febuxostat, and benzbromarone); Model 4 (primary model) further included serum calcium, vitamin D, alkaline phosphatase, C-reactive protein, and estimated glomerular filtration rate. eBMD: heel estimated bone mineral density; *β*: beta; *CI*: confidence interval.

A total of 424,105 UKB participants (54.84% female; mean age 56.73 ± 8.03 years) were included. The average serum urate level was 308.19 ± 80.07 μmol/L, and the mean eBMD was 0.54 ± 0.12 g/cm^2^. Baseline characteristics are detailed in Supplementary Table 1. After adjusting for covariates, four linear regression models revealed a significant positive association between serum urate and eBMD. In the primary Model 4, each one-unit increase in log-transformed serum urate was associated with a 0.008 g/cm² increase in eBMD (*β* = 0.008, 95% confidence interval [*CI*] = 0.006-0.011, *P* = 3.35 × 10^−10^) (Table 1). The positive association remained significant in sex-specific analyses, with stronger effects in females. Furthermore, a significant inverted U-shaped non-linear relationship was observed (*P*_non-linear_ < 0.001; Figure 2), with the positive association attenuated, even reversing, once serum urate exceeded 357 μmol/L (6 mg/dL), a threshold linked to potential hyperuricemia and elevated long-term gout risk [37].

**Table 1.** Phenotypic association between urate and eBMD.

Group	Model 1		Model 2		Model 3		Model 4	
	*β* (95% *CI*)	*P*-value	*β* (95% *CI*)	*P*-value	*β* (95% *CI*)	*P*-value	*β* (95% *CI*)	*P*-value
Total (*N* = 424,105)	0.026 (0.024–0.027)	1.80 × 10^−222^	0.010 (0.008–0.012)	2.18 × 10^−23^	0.011 (0.009–0.013)	1.52 × 10^−21^	0.008 (0.006–0.011)	3.35 × 10^−10^
Female (*N* = 232,559)	0.033 (0.031–0.035)	1.67 × 10^−242^	0.016 (0.013–0.018)	1.01 × 10^−35^	0.014 (0.012–0.017)	3.92 × 10^−23^	0.015 (0.012–0.019)	5.37 × 10^−20^
Male (*N* = 191,546)	0.027 (0.024–0.030)	1.14 × 10^−86^	0.014 (0.011–0.017)	1.81 × 10^−18^	0.015 (0.011–0.019)	1.16 × 10^−16^	0.006 (0.003–0.010)	1.37 × 10^−3^

Model 1 adjusted for age, sex (only the total participants), assessment centre, and the top 20 genetic principal components; Model 2 further adjusted for body mass index, education, Townsend deprivation index, income, smoking status, alcohol consumption, physical activity, and supplementation with calcium, vitamin D, and vitamin C; Model 3 further adjusted for hormone replacement therapy (only the female participants), disease status (diabetes, hypertension, hyperlipidemia) and medication use (including diphosphonates, corticosteroids, diuretics, beta-blockers, allopurinol, febuxostat, and benzbromarone); Model 4 further adjusted serum calcium, vitamin D, alkaline phosphatase, C-reactive protein, and estimated glomerular filtration rate. eBMD: heel estimated bone mineral density; *β*: beta; *CI*: confidence interval.

### 2.2. Genetic correlation

**Figure 3.**
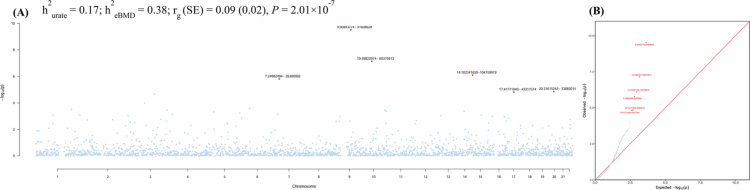
Global and local genetic correlations between urate and eBMD. (A) The Manhattan plot of the local genetic correlation from SUPERGNOVA, with the global genetic correlation from LDSC shown on the upper left. (B) The quantile-quantile (QQ) plot shows region-specific *P*-values for local genetic covariance. Red dots in the Manhattan plot and QQ plot denote loci exhibiting significant local genetic correlations after correction for multiple testing (*P* < 0.05/2218). eBMD: heel estimated bone mineral density; *h^2^*: heritability; *r_g_*: genetic correlation; SE: Standard error.

As shown in Figure 3, substantial heritability was observed for both urate (17%) and eBMD (38%). We found an overall shared genetic basis between urate and eBMD (*r*_*g*_ = 0.09, standard error = 0.02, *P* = 2.01 × 10^−7^).

Partitioning the whole genome into LD-independent regions, we identified six genomic regions exhibiting significant local genetic correlations between urate and eBMD (Figure 3). These regions, detailed in Supplementary Table 2, are located on chromosomes 7 (chr7: 24982494-26300992), 9 (chr9: 90893774-91608628), 10 (chr10: 59822974-60375613), 14 (chr14: 102341650-104759919), 17 (chr17: 41771040-43317574), and 20 (chr20: 31615242-33883015), each harbouring genes linked to urate or BMD. Notably, the most significant signal was at 9p24.3 (*P* = 3.06 × 10^−10^), harbouring *LINC02937* and *SYK* previously implicated in bone density [38]. The next strongest signals were at 10p15.3 (*P* = 7.35 × 10^−8^) and 14q11.1 (*P* = 8.19 × 10^−7^), containing *LINC01553* and ***MARK3***, respectively, which linked to both traits [20,39,40].

### 2.3. Cross-trait meta-analysis

**Figure 4.**
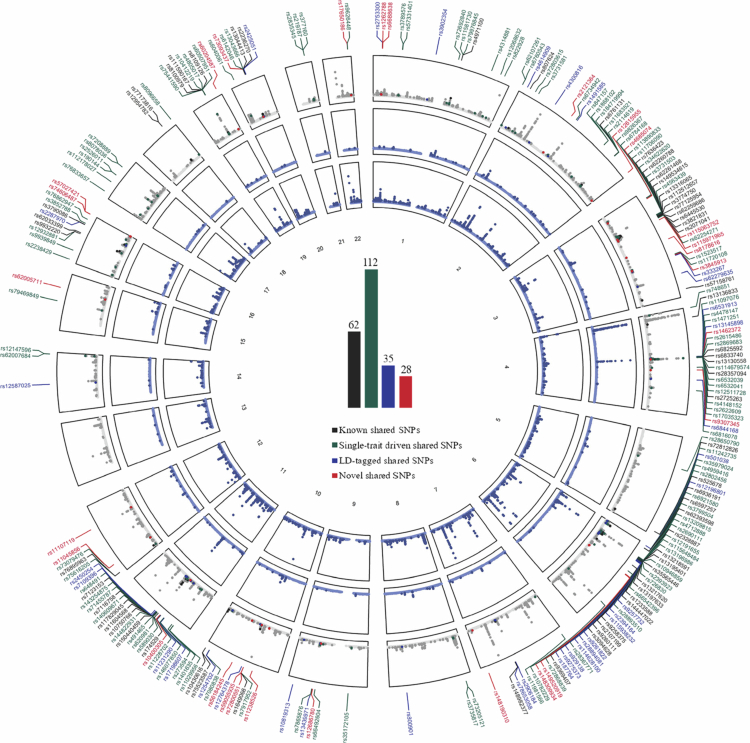
Pleiotropic loci between urate and eBMD identified from the cross-trait meta-analysis. The circular Manhattan plots displaying pleiotropic loci between urate and eBMD. The outermost circle shows the cross-trait meta-analysis results from CPASSOC. From the periphery to the centre, each circle shows the GWAS results for urate and eBMD. The dark grey/blue indicates variants with genome-wide significance (*P*_urate_ < 5 × 10^−9^ or *P*_eBMD_ < 6.6 × 10^−9^), while the light grey/blue indicates variants not reaching genome-wide significance. SNPs are classified into four categories based on their single-trait and cross-trait characteristics: (i) “Known” shared SNPs driven by both traits (*P*_urate_ < 5 × 10^−9^ and *P*_eBMD_ < 6.6 × 10^−9^); (ii) “Single-trait driven” shared SNPs driven by one of the two traits (either *P*_urate_ < 5 × 10^−9^ or *P*_eBMD_ < 6.6 × 10^−9^); (iii) “LD-tagged” shared SNPs not driven by both traits (*P*_urate_ ≥ 5 × 10^−9^ and *P*_eBMD_ ≥ 6.6 × 10^−9^) but in LD (r^2^ ≥ 0.2) with index SNPs identified in the original single-trait GWASs; (iv) “Novel” shared SNPs neither driven by both traits (*P*_urate_ ≥ 5 × 10^−9^ and *P*_eBMD_ ≥ 6.6 × 10^−9^) nor in LD (r^2^ < 0.2) with index SNPs identified in the original single-trait GWASs. These four categories of SNPs are represented in black, green, blue, and red, respectively, with their reference SNP ID (rsID) listed around the circles. The bar plot of pleiotropic loci is presented at the centre of the circle. eBMD: heel estimated bone mineral density; GWAS: genome-wide association study; LD: linkage disequilibrium; CPASSOC: cross-phenotype association; SNP: single-nucleotide polymorphism.

A total of 237 independent loci were identified as shared pleiotropic SNPs, all satisfying *P*_single-trait_ < 1 × 10^−5^ and *P*_CPASSOC_ < 5 × 10^−8^ (Figure 4). More details are provided in Supplementary Table 3, with annotations in Supplementary Table 4. These included 62 “known”, 112 “single-trait driven”, 35 “LD-tagged”, and 28 “novel” shared SNPs influencing both urate and eBMD. Notably, the most significant novel SNP, rs74606487 (*P*_CPASSOC_ = 2.22 × 10^−15^), is located at 16p13.3 near ***ZNF276***. The second most significant novel SNP, rs11045856 (*P*_CPASSOC_ = 5.22 × 10^−14^), is at 12p13.33 near *SLCO1B1*. Two novel SNPs, rs59055935 and rs72800551 (both at 10p15.3), map to ***BICC1***.

### 2.4. Fine-mapping and colocalization analysis

Fine-mapping of CPASSOC-identified shared SNPs yielded a 99% credible set of 5,318 candidate causal variants for urate and eBMD. More details are provided in **Supplementary Table 5**. Among these, 14 index SNPs had a 99% credible set containing only themselves, including three novel SNPs: rs149520919, rs72800551, and rs74606487, mapped to ***KHDRBS2***, ***BICC1***, and ***ZNF276***, respectively.

As shown in **Supplementary Table 6**, we found 35 shared loci that colocalized at the same candidate SNPs (*PPH4* > 0.90) for urate and eBMD. Among them, five index SNPs were novel: rs11238526, rs148190310, rs149520919, rs3845913, and rs8178616, mapped to *FXYD4*, *EZH2*, ***KHDRBS2***, *ADCY5*, and *PROS1*, respectively.

Notably, the novel shared SNP rs149520919 near ***KHDRBS2*** is particularly intriguing, as it was consistently captured across CPASSOC, fine-mapping, and colocalization analyses.

### 2.5. Gene-based analysis

We identified 872 non-duplicated genes for urate and 2,169 for eBMD **(Supplementary Tables 7-8)**. Merging these yielded 278 genes shared between urate and eBMD **(Supplementary Tables 9)**. Notably, seven genes—*PROS1*, *ADCY5*, *SLCO1B1*, ***RFT1***, *TMC4*, ***BICC1***, and ***ZNF276***—were mapped to novel shared SNPs identified by CPASSOC.

### 2.6. Transcriptome-wide association study

We identified multiple TWAS-significant gene-tissue pairs shared between urate and eBMD, suggesting gene-level genetic correlation across traits (**Supplementary Table 10**). In total, 150 unique genes were TWAS-significant for both traits, predominantly enriched in the tibial artery, fibroblasts, left ventricle, tibial nerve, and thyroid. Notably, three genes (***RFT1***, ***BICC1***, and ***ZNF276***) primarily enriched in brain regions (particularly the amygdala and caudate basal ganglia) were previously identified at novel shared SNPs by CPASSOC and MAGMA. These genes, implicated in both urate and eBMD [41–43], are functionally linked to the canonical Wnt signalling pathway [44–46], a key regulator of bone homeostasis [47] and neural development [48], implying a potential role for brain-skeleton interaction in the urate-eBMD relationship.

### 2.7. Functional enrichment and network analysis

**Figure 5.**
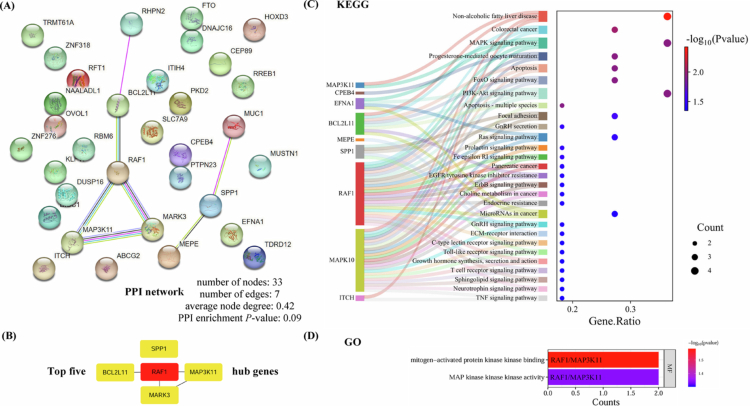
Functional enrichment and network analysis of shared genes identified by CPASSOC, MAGMA, and TWAS. (A) PPI network generated using the STRING database. Network nodes (3D bubbles) represent proteins, while edges represent protein-protein associations. Different coloured edges represent known, predicted, or other interactions. Coloured nodes represent query proteins and the first shell of interactors, while filled nodes represent a known or predicted 3D structure. (B) Schematic representation of the top five hub genes screened by the maximal clique centrality (MCC) from the PPI network. The colour gradient correlates with the rank according to the MCC: red indicates a higher rank, while yellow indicates a lower rank. (C) Sankey dot of KEGG pathway enrichment analysis after false discovery rate (FDR) correction. The left side of the figure shows a Sankey plot representing the genes included in each pathway. The right side displays a conventional bubble plot, where the size of each bubble corresponds to the number of genes in the pathway. (D) Bar plot with a colour gradient of GO biological function enrichment analysis after FDR correction. The colour of the bubble in (C) and the bar in (D) indicates the *P*-value, with red (blue) representing more (less) significant. PPI: protein-protein interaction; KEGG: Kyoto Encyclopaedia of Genes and Genomes; GO: Gene Ontology; MF: molecular function.

Functional enrichment and network analysis were conducted on 33 overlapping genes identified from CPASSOC, MAGMA, and TWAS (Supplementary Table 11). The PPI network comprised 33 nodes and 7 edges (Figure 5A). The top three interaction PPI pairs were ***MEPE-SPP1***, *RAF1-BCL2L11*, and *RAF1-MARK3*, with interaction scores of 0.92, 0.65, and 0.51, respectively (Supplementary Table 12). The top five hub genes of the PPI network were ***RAF1***, ***MARK3***, ***MAP3K11***, ***BCL2L11***, and ***SPP1*** (Figure 5B). KEGG pathway analysis highlighted several key pathways, including non-alcoholic fatty liver disease, colorectal cancer, MAPK signalling pathway, progesterone-mediated oocyte maturation, apoptosis, FoxO signalling pathway, and PI3K-Akt signalling pathway (Figure 5C). GO analysis identified two molecular functions, i.e., mitogen-activated protein kinase kinase binding and MAP kinase kinase kinase activity (Figure 5D). Full KEGG and GO enrichment results are provided in Supplementary Tables 13-14. These findings suggest that the identified genes may regulate both urate and eBMD through shared pathways and molecular functions, providing insights into their common genetic basis.

### 2.8. One-sample Mendelian randomisation

After excluding individuals with missing PRS data, 348,491 participants were included in the one-sample MR analysis. Baseline characteristics are shown in Supplementary Table 1. The PRS, constructed from 363 urate-associated variants, was strongly associated with serum urate (*β* = 0.049, 95% *CI* = 0.049–0.050, *P* < 0.001), explained 4.84% of the variance in urate (*R*^*2*^ = 0.048), and yielded an *F*-statistic of 153, supporting strong instrument strength (Supplementary Table 15). Associations between the PRS and selected baseline covariates are shown in Supplementary Table 16, with several covariates showing significant associations with the PRS, warranting cautious interpretation of the independence assumption. In addition, negative control outcome analyses using age and sex did not show significant associations with the PRS (all *P* > 0.05; Supplementary Table 17), providing no clear evidence of major residual bias.

**Figure 6.**
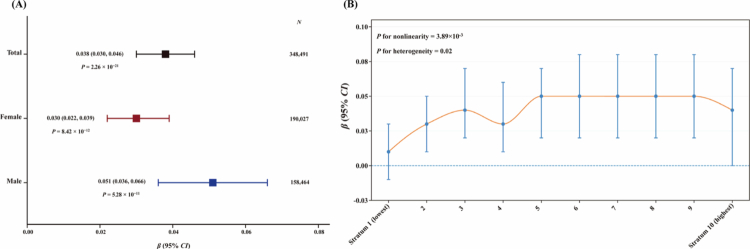
Linear and non-linear causal relationships between genetically predicted urate and eBMD in one-sample MR analyses. (A) Forest plot of the linear causal estimates for the associations of genetically predicted urate with eBMD in the total population and by sex. Squares represent the *β* estimates, and horizontal lines indicate the corresponding 95% *CIs*. (B) Non-linear one-sample MR analysis of genetically predicted urate and eBMD. Points represent the localised average causal effect estimates across strata, with vertical lines indicating 95% *CIs*. The orange curve represents the fitted non-linear trend, and the horizontal dashed line indicates *β* = 0. *P* values for non-linearity and heterogeneity are shown in the panel. The linear MR analysis was adjusted for age, sex (total participants only), assessment centre, genotyping batch, and the top 20 genetic principal components. The non-linear MR analysis further incorporated age-squared, age-by-sex interaction, and age-squared-by-sex interaction terms. eBMD, heel estimated bone mineral density; MR, Mendelian randomisation; *CI*, confidence interval; *β*, beta.

In the one-sample MR analysis, genetically predicted urate was positively associated with eBMD after adjustment for relevant covariates (*β* = 0.038, 95% *CI* = 0.030–0.046, *P* = 2.26 × 10^−21^; Figure 6A). This association remained significant in sex-specific analyses, with similar effect sizes in females and males (Figure 6A). In addition, evidence of a non-linear association between genetically predicted urate and eBMD was observed (*P*_non-linearity_ = 3.89 × 10^−3^). Evidence of heterogeneity across strata was also observed (*P*_heterogeneity_ = 0.02; Figure 6B). Stratum-specific estimates are presented in Supplementary Table 18.

## 3. Discussion

Our study provides the most comprehensive and systematic investigation of the phenotypic association and shared genetic architecture between urate and eBMD. We identified a significant phenotypic link underpinned by shared genetic bases, including vertical and horizontal pleiotropy, offering important implications for osteoporosis prevention and treatment.

Epidemiological investigations on the urate-eBMD relationship have been inconclusive. Using an enlarged dataset, we identified a positive association between urate levels in physiological states and eBMD, more pronounced in females, consistent with prior studies [6,18,19]. Notably, when serum urate levels exceeded 357 μmol/L (6 mg/dL), a threshold associated with hyperuricemia and elevated long-term gout risk [37], the positive association attenuated and even reversed. Similarly, a study of 182 ankylosing spondylitis patients revealed an inverted U-shaped urate-BMD relationship [49]. Altogether, these findings suggest that maintaining urate levels within a normal range (below 6 mg/dL) may benefit bone health.

The phenotypic link appears to be underpinned by a shared genetic basis. Despite a modest global genetic correlation (r_g_ = 0.09), significance was reinforced by six genomic regions harbouring genes implicated in urate or eBMD, validating our methodology and providing insights into shared mechanisms. Here, we highlight ***MARK3*** at 14q11.1, consistently identified across multiple analytical approaches. While *MARK3* has been implicated in both urate and eBMD, previous studies focused on each trait individually [11,39]. Our findings, however, provide compelling evidence that *MARK3* functions as a shared gene linking both traits. *MARK3*, a serine/threonine kinase first discovered within the *MARK* family, has been shown to increase bone mass by downregulating its expression to perturb cell signalling in osteoblasts [50]. Although the exact role of *MARK3* in urate metabolism remains unclear, the antioxidant effect of physiological urate on osteoblasts [5] points to the potential involvement of *MARK3* in the urate-eBMD relationship, a hypothesis that warrants further experimental validation.

The shared genetic basis may be attributable to causality (causal relationships) and/or pleiotropy (shared biological mechanisms). Our one-sample MR analysis provided robust evidence supporting a positive association between genetically predicted urate levels in physiological states and eBMD, consistent across genders. In contrast, previous MR studies using BMD or osteoporosis as outcomes [13–16] reported no associations, likely due to (i) augmented heritability of eBMD compared to osteoporosis or BMD at other bone sites, (ii) incorporation of updated GWAS data, and (iii) substantially enlarged number of IVs (363 vs. ≤ 26 in prior studies), enhancing statistical power and MR precision. Furthermore, adjustments for multiple confounders bolstered the robustness of our findings. Notably, our MR is the first to explore the non-linear causal relationship between urate and eBMD, with findings aligning with and further validating our observational results, underscoring the importance of maintaining higher urate levels within a normal range to support bone health.

To elucidate pleiotropy, we employed several advanced statistical genomics methods. CPASSOC identified 252 genes across 237 independent loci, including 24 genes at 28 novel loci. MAGMA uncovered 278 unique genes, while TWAS identified multiple gene-tissue pairs involving 150 independent genes. Together, these findings highlight the pervasive pleiotropy. Notably, SNP rs149520919 was identified as a potential causal variant shared between urate and eBMD, mapping to ***KHDRBS2*** and with robust evidence of colocalization (*PPH4* > 0.90). Existing literature links *KHDRBS2* solely to eBMD [43], and no study has explored its function in urate. Nevertheless, its homologue, *Sam68* (both members of the KH protein family known for RNA binding and regulation), is tyrosine phosphorylated in response to monosodium urate crystals [51] and acts as a negative regulator of osteoblast differentiation [52]. Given the inhibitory effect of excess urate on osteoblasts through inflammation and oxidative stress [5], we speculate that *KHDRBS2* may play a key role in the urate-eBMD relationship.

The 33 overlapping genes consistently identified across CPASSOC, MAGMA, and TWAS provide a molecular foundation for further mechanistic research. The PPI network analysis highlighted ***MEPE*** and ***SPP1*** as key interactors, both previously reported to be expressed in bone and cartilage, influencing extracellular matrix mineralisation and cell-matrix interactions [53]. Two of the top five hub genes (*MARK3* and *SPP1*) have been previously emphasised; thus, we focus on ***RAF1***, ***MAP3K11***, and ***BCL2L11***. Both *RAF1* and *MAP3K11* belong to the MAPK kinase family, while *BCL2L11* is a pro-apoptotic member of the BCL-2 protein family. Curcumin, a phenolic extract with anti-inflammatory, antioxidant, and anti-apoptotic properties, has been found to inhibit chondrocyte apoptosis by regulating the expression of BCL-2 and caspase-3 through the p38/MAPK pathway [54]. Given similar antioxidant properties, we hypothesise that urate may influence eBMD through a comparable mechanism. Furthermore, KEGG and GO analyses revealed several molecular pathways linking urate and eBMD, with a particular focus on the MAPK signalling pathway. Collectively, these findings underscore the essential roles of the MAPK pathway and apoptosis in the urate-eBMD relationship, which warrants further validation.

Our findings may carry potential clinical and public health implications. Firstly, maintaining higher urate levels within the normal range (below 6 mg/dL) appears to be a protective factor for eBMD. This suggests that simultaneous testing of urate and eBMD during early osteoporosis screening is crucial for early diagnosis, enabling precise management of urate levels to provide valuable support for clinical treatment. Secondly, the identification of shared genes and biological pathways between urate and eBMD paves the way for future mechanistic research, which could guide drug development and targeted therapies.

Several potential limitations should be acknowledged. **First**, our findings are restricted to individuals of European ancestry, limiting generalisability to other ethnic groups. **Second**, both the urate and eBMD GWAS summary statistics were derived from UKB participants, and overlap-related bias cannot be completely excluded. **Third**, although the PRS showed strong instrument strength, associations between the PRS and several baseline covariates warrant cautious interpretation of the independence assumption, and residual pleiotropy or other sources of bias cannot be entirely excluded. **Fourth**, due to the lack of GWAS data on hyperuricemia, we cannot determine whether the identified genes protect bone via antioxidant effects at normal urate levels or contribute to bone damage via inflammation and oxidative stress at elevated urate levels. **Fifth**, the observational analyses were based on a cross-sectional study design, which limits causal inference. **Finally**, the shared genes and relevant biological pathways identified in our study require further validation through in vitro or in vivo experiments to clarify their molecular roles in the urate-eBMD relationship.

## 4. Conclusions

In conclusion, our study establishes a clear link between urate and eBMD, providing evidence of phenotypic associations, genetic correlations, causal relationships, pleiotropic loci, shared genes, and gene-tissue pairs. These findings suggest that simultaneous testing of urate and eBMD during early osteoporosis screening is crucial for timely diagnosis and precise clinical management. Additionally, the shared genes and biological mechanisms identified may inspire future mechanistic research aimed at reducing osteoporosis risk and promoting bone health.

## Supplementary Material

Revised_Supplementary_Tables_Cleaned.xlsx Revised_Supplementary_Tables_Cleaned.xlsx

Supplementary Material Additional File 1

## Data Availability

This study did not generate new datasets or codes. It utilised individual cohort data of the UK Biobank participants (under Application #99713) and publicly available summary statistics. UK Biobank data access information is available at https://www.ukbiobank.ac.uk/, and GWAS summary statistics can be downloaded from the original GWASs or GWAS Catalogue (https://www.ebi.ac.uk/gwas/). Details on the analytical tools and codes used in this study can be found at the following sources: PLINK (https://www.cog-genomics.org/plink/1.9/), LDSC (https://github.com/bulik/ldsc), SUPERGNOVA (https://github.com/bulik/ldsc), CPASSOC (http://hal.case.edu/~xxz10/zhu-web/), FM-summary (https://github.com/hailianghuang/FM-summary), Coloc (https://chr1swallace.github.io/coloc/), VEP (https://grch37.ensembl.org/info/docs/tools/vep/), MAGMA (https://cncr.nl/research/magma/), FUSION (http://gusevlab.org/projects/fusion/), STRING (https://string-db.org/), OneSampleMR (https://github.com/remlapmot/OneSampleMR), and SUMnlmr (https://github.com/amymariemason/SUMnlmr).
